# Long live the kidney despite every threats: a case report

**DOI:** 10.11604/pamj.2025.51.35.47605

**Published:** 2025-06-09

**Authors:** Kubra Kaynar, Buse Misir, Huri Cihan Ataberk, Sevdegül Mungan, Ümit Çobanoğlu

**Affiliations:** 1 Department of Nephrology, Faculty of Medicine, Karadeniz Technical University, Trabzon, Turkiye; 2 Faculty of Medicine, İstanbul Medipol University, İstanbul, Turkiye; 3 Department of Internal Medicine, Faculty of Medicine, Karadeniz Technical University, Trabzon, Turkiye; 4 Department of Pathology, Faculty of Medicine, Karadeniz Technical University, Trabzon, Turkiye

**Keywords:** Carcinoma renal cell, carcinoma transitional cell, chronic renal insufficiency, case report

## Abstract

Kidneys are functionally affected by the diseases of other organs. Here, we present an elderly predialysis patient with a functioning kidney for 10 years of nephrological follow-up despite many comorbidities. A 75-year-old male patient with a medical history of hypertension for 15 years and 60 pack years of cigarette smoking, was diagnosed as muscle invasive bladder cancer (MIBC) and synchronous centrally located clear cell renal cell carcinoma (ccRCC) in the left kidney. At admission, the patient had an Estimated Glomerular Filtration Rate (eGFR) (CKD-EPI-cre) of 54mL/min/1.73m^2^, after left nephrectomy eGFR of the patient decreased to 35 mL/min/1.73m^2^. After ten years of follow-up, the patient's right kidney had been functional with a eGFR of 24 mL/min/1.73m^2^ despite radical cystectomy with urinary diversion, radical nephrectomy, heavy smoking, [cardiorenal syndrome, chronic obstructive pulmonary disease, and hypertension. It is well known that comorbidities such as hypertension, smoking, cancers, infections, pulmonary and heart diseases contribute to irreversible kidney damage and are additive to decreasing kidney function. Appropriate and early diagnosis and treatment of these comorbidities permit healthy aging of the kidneys without the need for dialysis.

## Introduction

The prevalence of clear cell renal cell carcinoma (ccRCC) which emerges from cortex and constitutes 70-80% of renal malign neoplasms differs between the countries and time periods [1]. The incidence of ccRCC is approximately 0.005% with a mortality rate of more than 40% [2]. In 2020 GLOBOCAN database, 573278 patients were diagnosed as bladder urothelial carcinoma (bUC) which is the 90% of all bladder cancers globally. Incidence of bladder and kidney cancers were reported as 3.1% and 2.2% respectively in the 2022 statistics. At the present, time, bladder cancers are reported as the sixth (after breast, prostate, lung, colorectal cancers and melanoma) most common cancer type worldwide, while kidney cancers are the seventh most common cancer type. These types of primary cancers are common seperately, however multiple primary cancers in one patient (especially co-occurrence of kidney and bladder cancer) have been very rarely reported [3]. As far as we know, 37 patients with dual cancers of bladder and kidney were reported till now [3].

The predialysis period has been defined for patients with chronic kidney disease (CKD) when their glomerular filtration rate (GFR) is lower than 30 mL/min/1.73m^2^ (approximately serum creatinine levels >2mg/dL). The predialysis nephrological care is very important for the delay of initiation of dialysis and lowering of mortality after dialysis [4]. The longest predialysis duration was declared as more than 72 months [4]. Here, we present a case who was simultaneously diagnosed as muscle invasive high grade papillary bUC and ccRCC and received predialysis nephrological care for 10 years with other comorbidities (chronic obstructive pulmonary disease and coronary artery disease).

## Patient and observation

**Patient information:** ten years ago, a 75-year-old male patient with a medical history of hypertension for 15 years and 60 pack years of cigarette smoking, applied to our department due to weakness.

**Clinical findings:** the patient's blood pressure was 140/70mmHg. His conjunctiva was pale. Body mass index was 22.1kg/m^2^.

**Figure 1:**
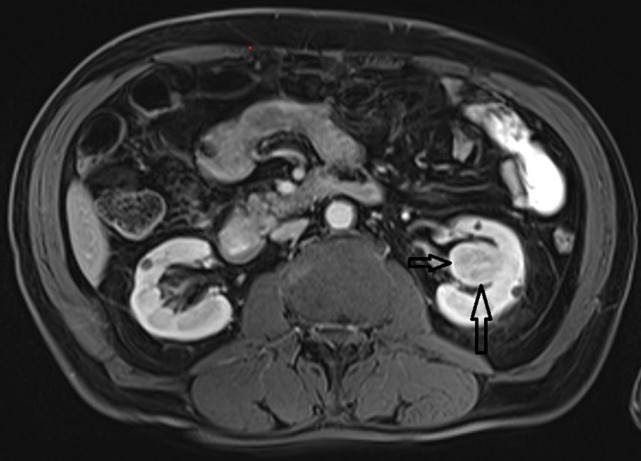
contrast enhancement in the left renal mass by magnetic resonance imaging

**Figure 2:**
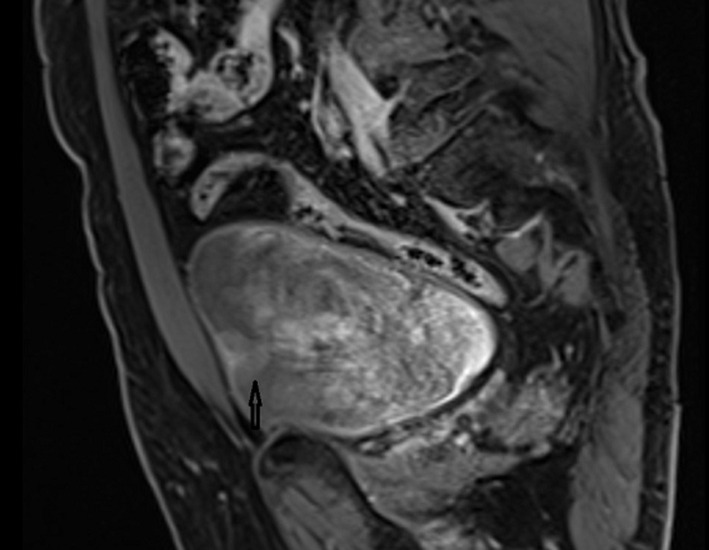
contrast enhancement in the polypoid lesion located in the bladder by magnetic resonance imaging

**Diagnostic assessment:** a decreased estimated glomerular filtration rate (eGFR) Chronic Kidney Disease Epidemiology Collaboration equation (CKD-EPI-cre based) of 54 mL/min/1.73m^2^, hematuria, anemia, left ventricular hypertrophy and a solid mass with a size of 24*23mm in the midportion of the left kidney were noticed. Further evaluations detected contrast enhancements in this renal mass and also in the polypoid lesion located in the bladder by magnetic resonance imaging (MRI) without distant metastasis (Figure 1, Figure 2).

**Diagnosis:** visible tumor biopsy, which revealed invasive high-grade bUC with muscle invasion was found in the bladder ceiling by cystoscopy. There was no distant and lymph node metastasis.

**Figure 3:**
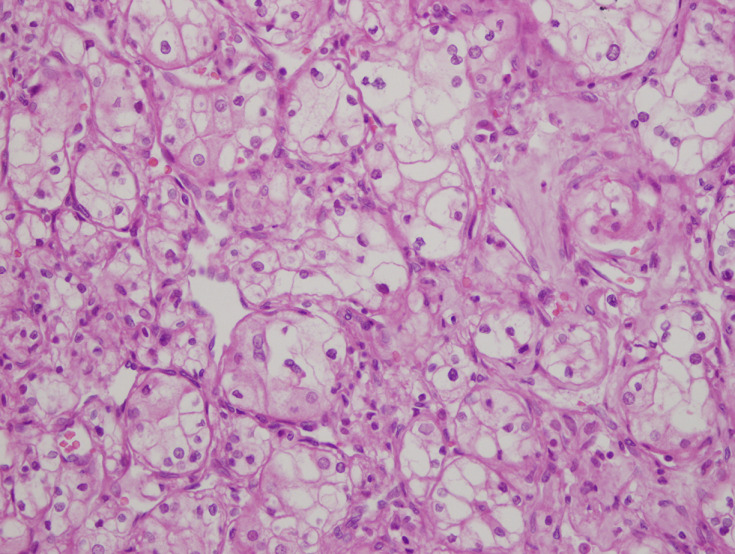
light microscopy findings showing clear cell renal cell carcinoma without necrosis, multifocality, perirenal fat, lymph node, and vessel invasion in nephrectomy material H&E×400

**Figure 4:**
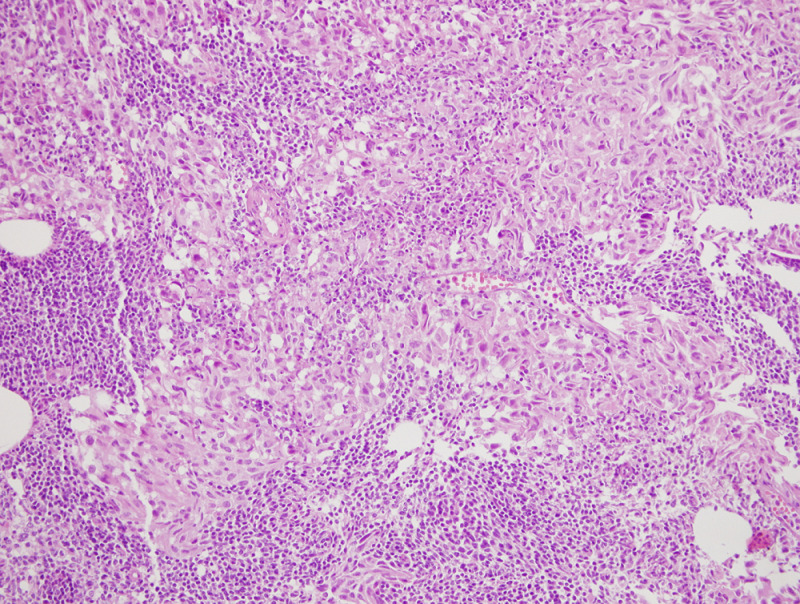
light microscopy findings showing muscle invasive high grade bladder urothelial cancer in cystectomy material H&Ex200

**Figure 5:**
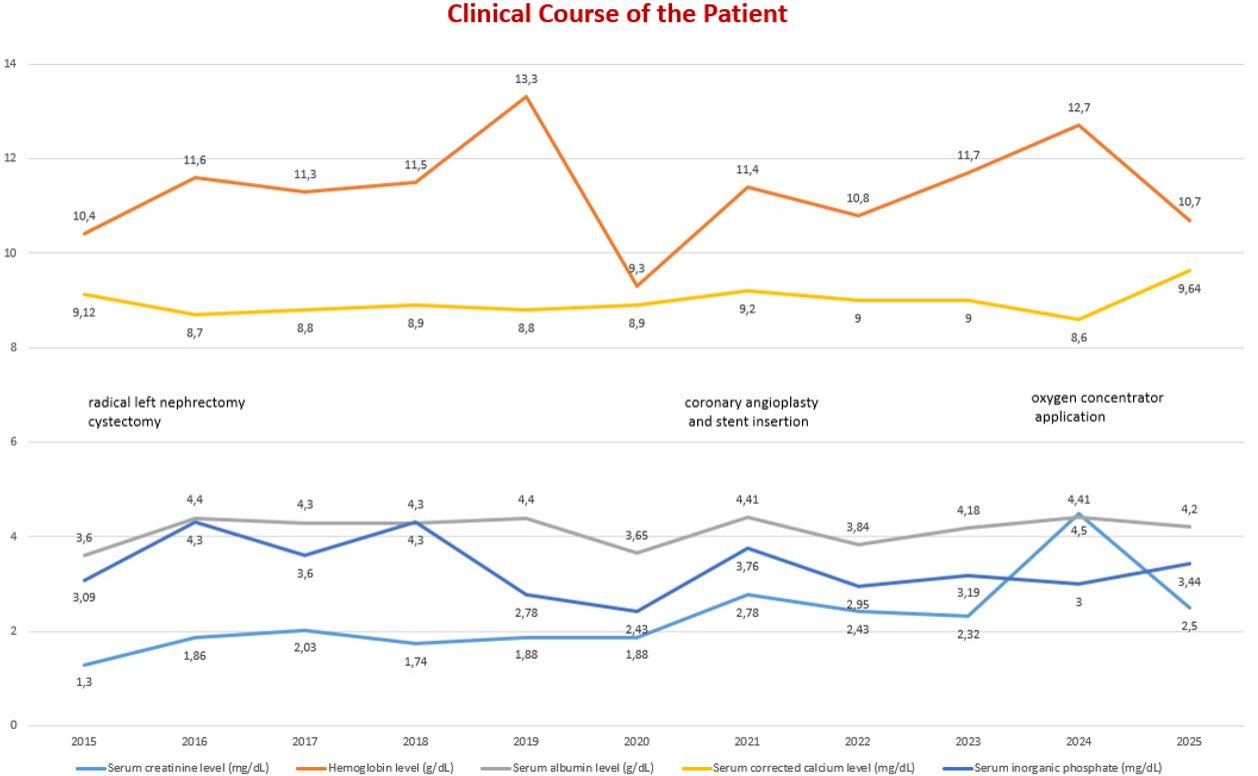
course of the patient's biochemical findings

**Figure 6:**
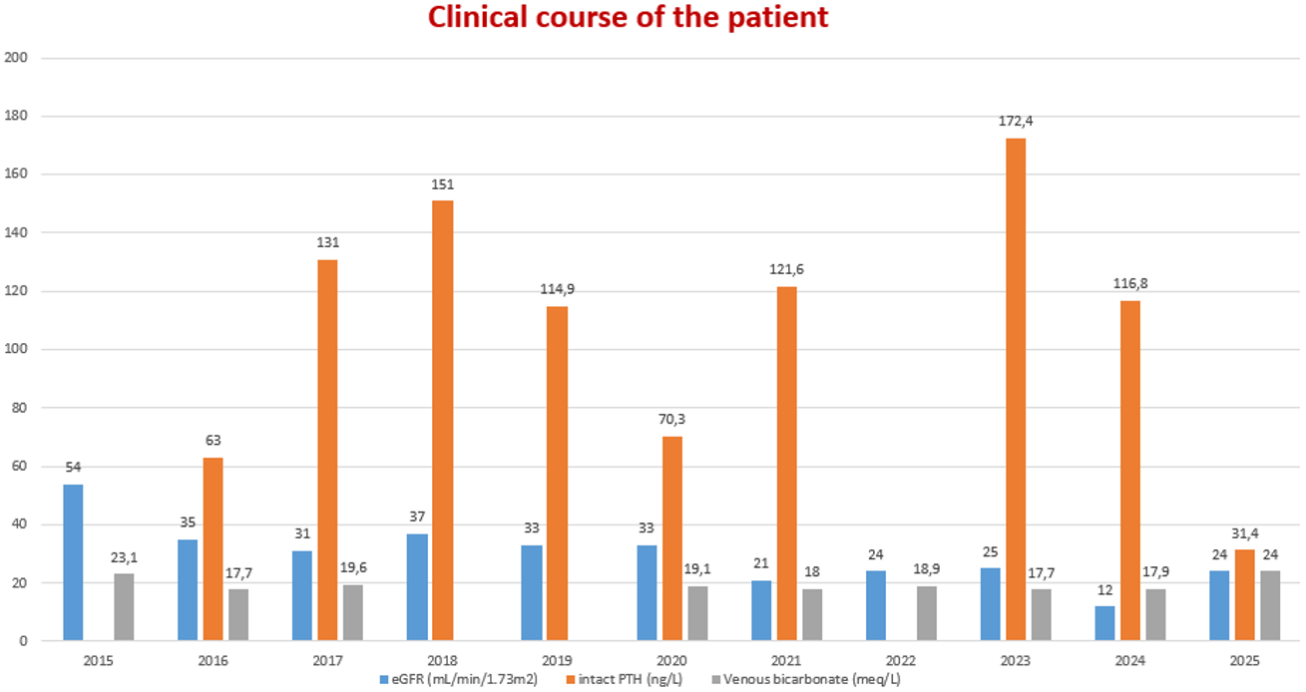
course of the patient's biochemical findings

**Therapeutic interventions:** the patient underwent laparotomy for radical left nephrectomy due to a tumor involving the central position of the left kidney, radical cystectomy, and prostatectomy. The pathology department reported a stage 1 ccRCC without necrosis, multifocality, perirenal fat, lymph node, and vessel invasion in nephrectomy material and muscle invasive high-grade bUC in cystectomy material (Figure 3, Figure 4). As the bladder was completely removed, ureterostomy was performed to help the right kidney drain urine. Colonoscopy which was performed to enlighten iron deficiency anemia, was normal. After left radical nephrectomy, kidney function further deteriorated with a eGFR (CKD-EPI-cre) of 35mL/min/1.73m^2^ (Figure 5, Figure 6). Diet (energy: 30kcal/kg/d, protein: 0.8-1g/kg/d, sodium 4-6g/d, low cholesterol), sodium bicarbonate (3g/d), allopurinol (150mg/d), atorvastatin (10mg/d), amlodipine (10mg/d), nebivolol (5mg/d), ferrous sulfate (325mg/d), vaccinations (pneumococcal, influenza), and hydration were prescribed to the patient. Chronic obstructive pulmonary disease (COPD) due to heavy smoking was also detected and dual bronchodilator therapy (glycopyrrolate-indacaterol) was given.

**Follow-up and outcome of interventions:** during routine follow-up, chronic fatigue and acute kidney injury episodes were noticed. Coronary angiography revealed severe (70-80%) luminal narrowings in the left anterior descending and right coronary arteries. Clopidogrel (75mg/d) and acetylsalicylate (100mg/d) were added to the patient's medications after balloon angioplasty and drug-eluting stent insertions. After this coronary intervention, kidney function improved. Three years later, his serum creatinine levels increased to 4.5mg/dL. At that time hypoxemia due to severe acute respiratory syndrome coronavirus 2 (SARS-CoV-2) infection was detected. The patient was hospitalized for nearly four weeks. At the time of discharge, his need for oxygen continued and long-term oxygen therapy was delivered via a home oxygen concentrator. When blood oxygen pressure (PaO2) and saturation (SaO2) got better, both his quality of life and kidney function also improved. His serum creatinine levels returned to a basal level of 2.5 mg/dL.

**Patient perspective:** he was shocked when he was diagnosed with kidney and bladder cancer 10 years ago. He had full family support, which eased his kidney and bladder losses. He became more carefull about his health and ceased smoking. Now, he is happy with his family, has accepted his diseases, takes his medicines and goes for check-ups regularly.

**Informed consent:** written informed consent for publication of his medical report, and for all the interventions was obtained from the patient.

## Discussion

The second most common urological malignancy is bUC globally. The standard care for patients with muscle-invasive bladder cancer (MIBC) who are generally fit for operation is radical cystectomy (removal of the bladder with extended pelvic lymph nodes dissection with or without removal of internal genital organs) with urinary diversion (reconstruction of the lower urinary tract) [5]. Although radical cystectomy is endorsed for patients with MIBC, preoperative presence of CKD progresses to advanced stages postoperatively [6]. Nevertheless, this approach increased 5-year MIBC-specific survival rates up to 76%. Urinary reconstructive options after radical cystectomy are either ileal conduit or cutaneous ureterostomy. Although similar survival rates were found, the complications were reported to be lower among patients whose urinary diversion was cutaneous ureterostomy than those with ileal conduit. Even though the effect of RC on kidney functions depends on type of the urinary diversion and preoperative presence of diabetes, CKD, radiation therapy, age, and hypertension, it was reported that postoperative 5 year-decline in the eGFR of patients with RC and ileal conduit was 1.74mL/min/1.73m^2^ [6]. Another retrospective study performed in 70 elderly patients with RC and cutaneous ureterostomy found that preoperative eGFR of 74.3mL/min/1.73m^2^ declined to 54.6mL/min/1.73m^2^ after 6 months postoperatively [7].

Among all of the cancers, RCC represents 3% of them. The established risk factors for etiology of RCC are smoking, obesity, hypertension and genetic susceptibility. Our patient had several of them such as smoking and hypertension. Treatment option for centrally located stage 1 RCC is radical nephrectomy which was performed in our patient [1]. A study conducted in a total of 202 patients who underwent radical nephrectomy found that preoperative mean eGFR of 84.9 mL/min/1.73m^2^ declined to 60.3mL/min/1.73m^2^ postoperatively [8]. Similarly, our patient's preoperative eGFR of 54mL/min/1.73m^2^ decreased to 35mL/min/1.73m^2^ after nephrectomy.

Cardiac diseases lead to kidney dysfunction, both reducing renal perfusion and increasing congestion in the kidneys. It was also reported that patients with non-ST-segment elevation myocardial infarction and reduced kidney function receive less invasive management than those with preserved kidney function, causing worse cardiac and renal outcomes in patients with impaired kidney function [9]. Our patient's kidney function improved after coronary intervention for clogged arteries.

Impaired gas exchange in the lungs also led to reduced GFR, renal blood flow, sodium and water excretion. Kidney dysfunction is both a complication and a negative prognostic factor for patients with COPD exacerbation [10]. Our patient had an acute COPD exacerbation after SARS-CoV-2 infection. Eventually, the eGFR of the patient deteriorated further. Oxygen therapy improved the kidney function of the patient. Aging also decreases renal functional reserve in our patient, apart from these comorbidities. As a result, optimal management of patients with CKD includes prevention of infections, cessation of smoking, treatment of hypertension, hyperlipidemia, anemia, fluid depletion, fluid overload, and comorbidities.

## Conclusion

It is well known that comorbidities such as hypertension, smoking, cancers, infections, pulmonary and heart diseases contribute to irreversible kidney damage and are additive to decreasing kidney function. Appropriate and early diagnosis and treatment of these comorbidities permit healthy aging of the kidneys without the need for dialysis.
